# Association of Life-Course Neighborhood Deprivation With Frailty and Frailty Progression From Ages 70 to 82 Years in the Lothian Birth Cohort 1936

**DOI:** 10.1093/aje/kwac134

**Published:** 2022-07-27

**Authors:** Gergő Baranyi, Miles Welstead, Janie Corley, Ian J Deary, Graciela Muniz-Terrera, Paul Redmond, Niamh Shortt, Adele M Taylor, Catharine Ward Thompson, Simon R Cox, Jamie Pearce

**Keywords:** aging, frailty, life-course approach, neighborhood deprivation, structured life-course modeling

## Abstract

Neighborhood features have been postulated to be key predictors of frailty. However, evidence is mainly limited to cross-sectional studies without indication of long-term impact. We explored how neighborhood social deprivation (NSD) across the life course is associated with frailty and frailty progression among older Scottish adults. Participants (*n* = 323) were persons selected from the Lothian Birth Cohort 1936 with historical measures of NSD in childhood (1936–1955), young adulthood (1956–1975), and mid- to late adulthood (1976–2014). Frailty was measured 5 times between the ages of 70 and 82 years using the Frailty Index. Confounder-adjusted life-course models were assessed using a structured modeling approach; associations were estimated for frailty at baseline using linear regression and for frailty progression using linear mixed-effects models. Accumulation was the most appropriate life-course model for males; greater accumulated NSD was associated with higher frailty at baseline (*b* = 0.017, 95% confidence interval: 0.005, 0.029). Among females, the mid- to late adulthood sensitive period was the best-fitting life-course model, and higher NSD in this period was associated with widening frailty trajectories (*b* = 0.005, 95% confidence interval: 0.0004, 0.009). To our knowledge, this is the first investigation of the life-course impact of NSD on frailty in a cohort of older adults. Policies designed to address deprivation and inequalities across the full life course may support healthy aging.

## Abbreviations

CI: confidence interval
DAG: directed acyclic graph
IQ: intelligence quotient
LARS: least angle regression
LASSO: least absolute shrinkage and selection operator
LBC1936: Lothian Birth Cohort 1936
NSD: neighborhood social deprivation
OSC: occupational social class

The world’s population is rapidly aging, resulting in growing numbers of older adults and a greater proportion of the population aged 60 years or more. Whereas population aging is most advanced in high-income countries (1), changes to the demographic profile can be observed worldwide (2). In 2017, there were 962 million individuals aged ≥60 years; that number is expected to double by 2050, with an anticipated 3-fold rise among the oldest old (≥80 years) (2). Not everyone ages in the same way: Age-related decline in physical and mental capacities and functional abilities is not homogenous (3).

Frailty is an age-related syndrome characterized as increased vulnerability, loss of resistance to stressors, and decreased reserves of capacity, which develops as a consequence of cumulative declines in several interrelated physiological systems (4, 5). Frailty among older people heightens the risk of falls (6), morbidity (4), and mortality (7); it is linked to increased health and social-care costs (8). The prevalence of frailty varies considerably, with higher rates among females (9, 10), the socioeconomically disadvantaged, and ethnic minority groups (8). Similarly, a range of sociodemographic (e.g., socioeconomic status) and lifestyle (e.g., physical activity) factors are associated with frailty progression (11).

Despite growing interest in the contextual determinants of various aspects of health and well-being (12, 13), there is an important research gap in understanding how environments—particularly local, neighborhood-level factors—contribute to frailty (3). In a recent review, Fritz et al. (3) found that social and physical characteristics of neighborhoods, including deprivation, ethnic diversity/heterogeneity, social cohesion, and walkability, are associated with frailty. However, the evidence relied heavily on cross-sectional data, and none of the identified investigations utilized repeated neighborhood assessments through time (3). Similarly, little is known about how neighborhood-level factors are associated with frailty progression (3, 11). Places evolve over time (e.g., urban redevelopment, economic decline); depending on the timing of exposure during human development, neighborhood features may have a differential and long-lasting impact on health (12). Describing how living in different contexts across the life course is associated with frailty is crucial in identifying modifiable risk factors, understanding age-related decline, and developing age-friendly policies to support healthy aging.

Using rarely available longitudinal individual- and area-level data, we aimed to fill this research gap by applying the life-course framework. First, using a structured approach (14), we identified the most appropriate life-course model(s) for frailty at age 70 years. We considered sensitive periods (i.e., whether the association between neighborhood and frailty is stronger during particular developmental periods), accumulation (i.e., whether the sum of exposures over time is associated with frailty), and effect modification (i.e., whether the impact of neighborhood on frailty is modified by exposure in an earlier period). Second, we estimated the associations between best-fitting model(s) and frailty at age 70 years and frailty progression between the ages of 70 and 82 years.

## METHODS

### Study sample

Data were drawn from the Lothian Birth Cohort 1936 (LBC1936), a cohort of relatively healthy older adults residing in Edinburgh and the Lothian region of Scotland, United Kingdom. Participants were born in 1936 and recruited between 2004 and 2007, with a mean age of 70 years (15). The cohort (*n* = 1,091 at wave 1) has been followed up at ages 73 years (*n* = 866), 76 years (*n* = 697), 79 years (*n* = 550), and 82 years (*n* = 431), with attrition mainly due to withdrawal (including from illness) and death (15). A unique feature of the LBC1936 is the availability of validated cognitive ability test scores recorded at age 11 years, since cohort members participated in the Scottish Mental Survey 1947, a nationwide intelligence test of all 11-year-olds carried out on June 4, 1947 (15).

### Measures

#### Neighborhood social deprivation.

Historical residential addresses were collected retrospectively for the LBC1936 as part of a “life grid” questionnaire administered in 2014 (mean age = 78 years), an established and validated way of gathering information on life-course circumstances (16). With major historical events (e.g., the Falklands War of 1982) serving as memory prompts, surviving participants were asked to recall their home addresses for every decade of their lives (12, 15). Out of 704 contacts, 593 provided 7,423 addresses, which were geocoded using automatic geocoders (i.e., Nominatim (https://nominatim.org/), Google's geocoder (Google, Inc. (Mountain View, California))) and historical building databases (12).

Information on neighborhood social deprivation (NSD) for residents of the City of Edinburgh was captured once per decade during participants’ lives. Between 1941 and 1971, this was done using a historical index of multiple types of deprivation (i.e., population density, overcrowding, infant mortality, tenure (percentage of households renting their accommodations), and amenities) (12), and between 1981 and 2011 with the Carstairs index of deprivation (i.e., male unemployment, overcrowding, car ownership, and social class) (17). All data were aggregated to 1961 census ward boundaries (*n* = 23) to ensure consistent spatial units across time, which was required in order to compute missing data for some historical indicators (details are presented elsewhere) (12). Comparability across decades was maintained by computing deprivation index *z* scores (12). NSD indices were linked to participants’ residential histories using 10-year time periods (e.g., 1941 NSD was linked to addresses from 1936–1945). We explored correlations between individual NSD scores per decade (see Web Figure 1, available at https://doi.org/10.1093/aje/kwac134) and computed average exposure in childhood (1936–1955; age ≤19 years), young adulthood (1956–1975; ages 20–39 years), and mid- to late adulthood (1976–2014; ages 40–78 years); these measures were computed for participants for whom at least 1 Edinburgh-based address was reported in all periods.

#### Frailty Index.

Frailty was assessed across all follow-up waves utilizing the Frailty Index, a continuous measure representing frailty as an accumulation of health deficits (e.g., symptoms, diagnosis, impairments) across multiple body systems (18, 19). As an objective marker of deficit accumulation, the Frailty Index is applicable in every individual regardless of disability status. It indicates the number of clinical conditions present and provides a useful measure for assessing frailty trajectories over time (20). Following recommended guidelines (18), we extracted information for 30 deficits covering physical, psychological, and cognitive systems on which data are routinely collected for LBC1936 waves (15). Cutoff scores indicating the presence (value = 1) or absence (value = 0) of deficits were previously established for the cohort (21) (see Web Table 1). Frailty scores were calculated by summing each participant’s deficits and dividing by the total number of possible deficits (*n* = 30). The indicator ranged between 0 and 1, with higher values showing higher degrees of frailty.

#### Covariates.

Life-course covariates were identified on the basis of the literature (12, 21, 22) and incorporated into a directed acyclic graph (DAG) taking into consideration time-specific confounding and selection into similar neighborhoods (Web Figure 2). Variables included sex, age (in years; time-variant), parental occupational social class (OSC) (professional-managerial (I/II) vs. skilled, partly skilled, or unskilled (III/IV/V)) (23), childhood intelligence quotient (IQ) score at age 11 years (as measured with the Moray House Test (15)), duration of full-time education (years), and childhood smoking (initiation of smoking before age 16 years). Data on adult OSC (I/II vs. III/IV/V) (23) and current smoking status (yes, no) were extracted at age 70 years.

### Statistical analysis

We compared study variables between included participants and the rest of the baseline sample to investigate sample bias, with differences tested using 2-sample *t* tests and χ^2^ tests. To explore the life-course associations between NSD and frailty and frailty progression, we applied the modified 2-stage structured life-course modeling approach (14) originally proposed by Mishra et al. (24). Analyses were conducted in R 4.0.3 (R Foundation for Statistical Computing, Vienna, Austria) (25), separately for men and women (9–11).

In stage 1, the most appropriate life-course models for frailty at age 70 years were identified using the least angle regression (LARS) algorithm for variable selection. LARS provides a structured and unbiased way to select an input variable (or a combination) from multiple simultaneously competing ones with the strongest association with the outcome (14). It implements the least absolute shrinkage and selection operator (LASSO) to identify the best-fitting variable (14, 26); after the first variable is selected, the procedure identifies the combination of 2 variables explaining the largest outcome variance, and so on until all input variables are selected. Following recommended practice (27), we used the elbow plot depicting the proportion of outcome variance (*R*^2^) explained by the selected variable(s) (14) and the covariance test for the LASSO indicating improvement in the explained outcome proportion (*P* < 0.05) (28).

Six competing life-course models were encoded as LARS input variables. Sensitive periods were captured as NSD in childhood, young adulthood, and mid- to late adulthood; accumulation was the average exposure across these. Effect modifications in early and later life were operationalized as interactions between childhood and young adulthood NSD and between young adulthood and mid- to late adulthood NSD (we added 10 to each observation to avoid negative values during multiplication). To account for confounding, we regressed input variables on covariates identified as common confounders across all life-course models (age, parental OSC) and entered the model residuals into LARS (14).

In stage 2, we estimated the effect size of selected models in a multiple regression framework separately for age 70 frailty and frailty progression between ages 70 and 82 years. Three sets of confounders were considered relevant for the proposed life-course models (see DAG (Web Figure 2)):

- *Model 1*: Age and parental OSC—common confounders for all life-course models and considered to be the most appropriate adjustment factors for the childhood sensitive period.
- *Model 2*: Model 1 + duration of education, childhood smoking, and IQ at age 11 years—relevant confounders for the young adulthood sensitive period and early-life effect modification.
- *Model 3*: Model 2 + adult OSC and current smoking—relevant for accumulation, the mid- to late adulthood sensitive period, and later-life effect modification.

Where applicable, we also added NSD from the previous life-course period to account for selection into similar neighborhoods (29). Calculations for frailty were based on linear regression. Frailty progression was fitted in linear mixed-effects regression with random intercepts and slopes—chosen as the best-fitting model—where the NSD × age interaction represented change in frailty scores. Coefficients (*b*) with 95% confidence intervals (CIs) based on scaled and mean-centered continuous variables were calculated; we also report fully standardized coefficients (β) to aid interpretation of effect sizes. After fitting linear mixed-effects models for frailty progression, we calculated Johnson-Neyman intervals with adjustment for false-discovery rates to explore where NSD slopes changed between regions of significance and nonsignificance conditional on age. Given the limitation of our overall framework (i.e., sex-stratified analyses), we performed confirmatory analyses by testing the sex × NSD interaction in the total sample.

We conducted 6 sets of sensitivity analyses. First, instead of using common confounders (i.e., age, parental OSC) to produce model residuals for LARS, we regressed life-course models on their specific confounders. Second, instead of including participants who had at least 1 Edinburgh-based address in childhood, young adulthood, and mid- to late adulthood, we reran analyses with participants who remained in Edinburgh throughout their lives. Third, we considered NSD measures only until 2005, to avoid temporal overlap between exposure and outcome assessment (2004/2007–2017/2019). Fourth, we tested linearity of associations by replacing continuous NSD variables with categorical ones indicating low, moderate, and high deprivation. Fifth, to facilitate the clinical interpretation of findings, we replaced the continuous Frailty Index by a binary indicator using the cutpoint of 0.2, which distinguishes between robust individuals and those approaching a frail state (18); models were based on (mixed-effects) logistic regression with parameter estimates expressed as odds ratios. Last, we excluded participants with cognitive impairment at the time of residential history data collection (*n* = 10), in order to reduce the risk of including inaccurate addresses in our analyses. Cognitive impairment was defined as either having a diagnosis of dementia or scoring less than 24 points on the Mini-Mental State Examination (30) during waves 1–4.

#### Ethics approval.

The LBC1936 Study was conducted according to the Declaration of Helsinki guidelines. Ethical permission was obtained from the Multi-Centre Research Ethics Committee for Scotland, the Lothian Research Ethics Committee (wave 1), and the Scotland A Research Ethics Committee (waves 2–5). Written consent was obtained from all participants.

## RESULTS

**Figure 1:**
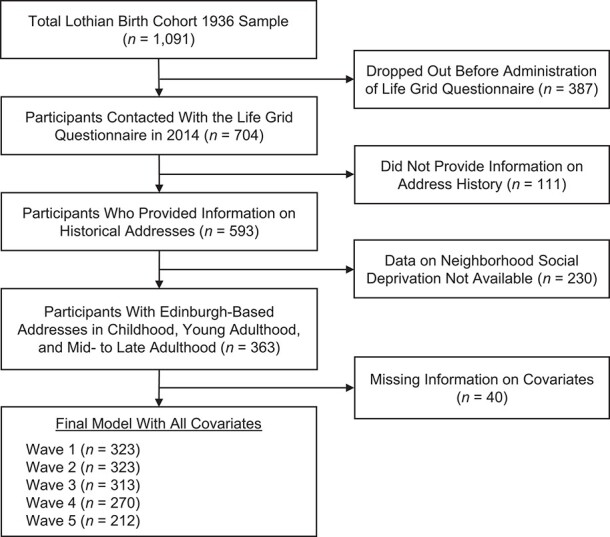
Selection of members of the Lothian Birth Cohort 1936 for a study of neighborhood social deprivation and frailty among Scottish older adults, 1936–2019.

From the total LBC1936 sample, we included 323 individuals in our analyses; 35% of the original sample dropped out before residential history information was collected in 2014, and 21% did not have Edinburgh-based addresses at least once in all 3 developmental periods, precluding linkage to information on neighborhood deprivation (Figure 1). Included participants were younger, on average, and less likely to smoke or be frail at age 70 years. In the analytical sample, there were 161 men and 162 women; women had a higher IQ at age 11 years and were less likely to smoke before age 16 years. Participants’ exposure to socially deprived neighborhoods decreased during the course of their lives, while their frailty increased across waves (Table 1).

**Table 1:** Characteristics of Lothian Birth Cohort 1936 Members Included in and Excluded From a Study of Neighborhood Social Deprivation and Frailty, 1936–2019

	**Inclusion Status**										**Analytical Sample** ^**a**^						
	**Total (*n* = 1,091)**			**Excluded (*n* = 768)**			**Included (*n* = 323)**				**Male (*n* = 161)**			**Female (*n* = 162)**			
**Characteristic**	**No.** ^**c**^	**%**	**Mean (SD)**	**No.** ^**c**^	**%**	**Mean (SD)**	**No.**	**%**	**Mean (SD)**	***P* Value** ^**b**^	**No.**	**%**	**Mean (SD)**	**No.**	**%**	**Mean (SD)**	***P* Value** ^**b**^
Age, years	1,091		69.53 (0.83)	768		69.62 (0.86)	323		69.34 (0.74)	<0.001	161		69.33 (0.75)	162		69.35 (0.73)	0.853
Sex																	
Male	548	50.23		387	50.39		161	49.85									
Female	543	49.77		381	49.61		162	50.15		0.922							
Parental occupational social class																	
I and II	260	27.08		172	22.40		88	27.24			44	27.33		44	27.16		
III, IV, and V	700	72.92		465	60.55		235	72.76		0.997	117	72.67		118	72.84		1.000
IQ at age 11 years	1,028		100.00 (15.00)	705		99.44 (15.31)	323		101.23 (14.22)	0.068	161		99.56 (15.01)	162		102.89 (13.23)	0.035
Duration of education, years	1,091		10.74 (1.13)	768		10.78 (1.16)	323		10.64 (1.06)	0.058	161		10.66 (1.04)	162		10.62 (1.07)	0.727
Childhood smoking																	
Yes	226	20.71		166	21.61		60	18.58			44	27.33		16	9.88		
No	865	79.29		602	78.39		263	81.42		0.294	117	72.67		146	90.12		<0.001
Adult occupational social class																	
I and II	592	55.33		413	53.78		179	55.42			82	50.93		97	59.88		
III, IV, and V	478	44.67		334	43.49		144	44.58		1.000	79	49.07		65	40.12		0.132
Current smoking																	
Yes	125	11.46		101	13.15		24	7.43			10	6.21		14	8.64		
No	966	88.54		667	86.85		299	92.57		0.009	151	93.79		148	91.36		0.535
Frailty Index^d^																	
Wave 1	1,091		0.16 (0.09)	768		0.17 (0.09)	323		0.14 (0.07)	<0.001	161		0.14 (0.08)	162		0.15 (0.07)	0.757
Wave 2	866		0.18 (0.09)	543		0.18 (0.09)	323		0.17 (0.08)	0.014	161		0.17 (0.08)	162		0.17 (0.07)	0.568
Wave 3	697		0.20 (0.09)	384		0.21 (0.10)	313		0.20 (0.08)	0.215	157		0.20 (0.08)	156		0.20 (0.08)	0.950
Wave 4	550		0.21 (0.09)	280		0.22 (0.09)	270		0.21 (0.09)	0.172	128		0.21 (0.09)	142		0.21 (0.09)	0.908
Wave 5	431		0.22 (0.09)	219		0.22 (0.09)	212		0.21 (0.09)	0.186	94		0.21 (0.09)	118		0.21 (0.09)	0.833
Neighborhood social deprivation score																	
Childhood							323		0.41 (3.29)		161		0.59 (3.45)	162		0.24 (3.13)	0.337
Young adulthood							323		−0.64 (2.61)		161		−0.49 (2.78)	162		−0.79 (2.42)	0.303
Mid- to late adulthood							323		−1.95 (2.78)		161		−1.99 (2.87)	162		−1.91 (2.70)	0.797

Abbreviations: IQ, intelligence quotient; SD, standard deviation.

^a^ Subsamples were part of the included sample.

^b^ *P* values were based on 2-sample *t* tests for mean differences and on χ^2^ tests for differences in distribution.

^c^ Numbers in subgroups may not sum to totals because of missing values.

^d^ Numbers differ across waves because of loss to follow-up.

**Figure 2:**
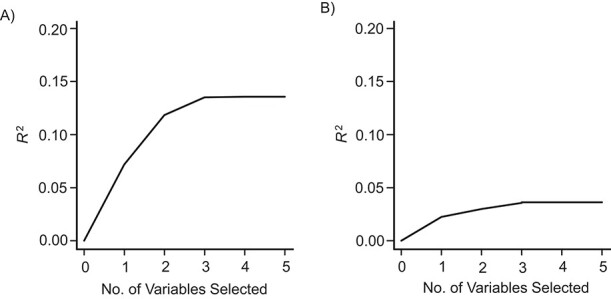
Least angle regression (LARS) selection procedure used for life-course models of neighborhood social deprivation based on explained outcome variance (i.e., frailty at age 70 years), Lothian Birth Cohort 1936, 1936–2019. LARS input variables were residuals of variable-encoded life-course models, regressed on age and parental occupational social class as common confounders. The LARS procedure first selects the variable with the largest outcome variance explained, followed by a combination of additional variables with increasingly strong associations with the outcome. Models were fitted separately for males and females. For males (A), the selection steps were 1) accumulation, 2) + mid- to late adulthood sensitive period (SP), 3) + childhood SP, 4) + early-life effect modification, and 5) + later-life effect modification; for females (B), the selection steps were 1) mid- to late adulthood SP, 2) + young adulthood SP, 3) + early-life effect modification, 4) + childhood SP, and 5) + later-life effect modification. Young adulthood SP for males and accumulation for females were dropped because of collinearity.

Among males, the LARS procedure identified accumulation as the best-fitting life-course model, accounting for 7.16% of the unexplained variance (*P* < 0.001). Although the elbow plot (Figure 2A) indicated further improvements by adding mid- to late adulthood and childhood sensitive periods (*R*^2^ = 0.135), these steps were not supported by the covariance test of LASSO (*P* = 0.368). Among females (Figure 2B), the mid- to late adulthood sensitive period was the first selected model (*R*^2^ = 0.022); however, it was not supported by the covariance test (*P* = 0.087).

**Figure 3:**
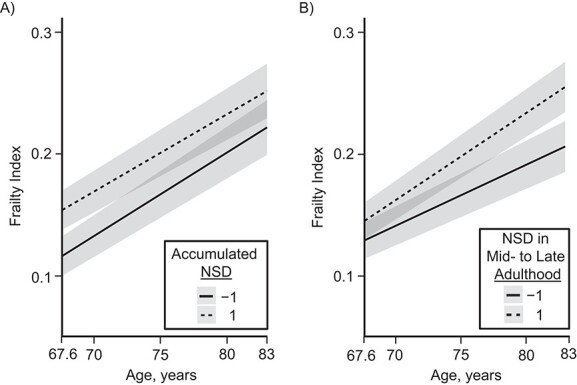
Frailty progression among males (A) and females (B) according to selected neighborhood social deprivation (NSD) life-course models, Lothian Birth Cohort 1936, 1936–2019. The plots show predicted probabilities with discrete predictors held constant at their proportions. Prediction lines represent ±1 standard deviation of NSD; the gray shaded areas show 95% confidence intervals. Calculations were based on the most appropriate life-course models for male (i.e., accumulation) and female (i.e., mid- to late adulthood sensitive period) participants.

After choosing the best-fitting life-course models, we first estimated the association between NSD and frailty at baseline. For both selected models, full adjustment models (model 3) were deemed most appropriate on the basis of our DAG. Among men, a 1–standard-deviation higher score in accumulated NSD was associated with a 0.017-point (95% CI: 0.005, 0.029) higher value in the Frailty Index score at age 70 years, presenting a moderate effect size (β = 0.223) (Table 2). Post hoc linear regression adjusted for false-discovery rate explored periods most likely contributing to accumulation: Childhood NSD (model 1: *b* = 0.021 (95% CI: 0.009, 0.031); adjusted *P =* 0.027) and mid- to late adulthood NSD (model 3: *b* = 0.015 (95% CI: 0.002, 0.027); adjusted *P* = 0.029) were associated with frailty, pointing towards relaxed accumulation (i.e., periods are not contributing equally to the risk) among males (31). Among females, mid- to late adulthood NSD was not associated with frailty at baseline (*b* = 0.010; 95% CI: –0.002, 0.022). In the total sample, sex differences were present for all reported associations (*P* < 0.05).

**Table 2:** Associations Between Neighborhood Social Deprivation, Frailty, and Frailty Progression in Lothian Birth Cohort 1936, 1936–2019^a^

	**Sex and Most Appropriate Life-Course Model**							
	**Male (*n* = 161): Accumulation**				**Female (*n* = 162): Mid- to Late Adulthood Sensitive Period** ^**b**^			
**Covariate**	***b***	**95% CI**	***P* Value**	**β**	***b***	**95% CI**	***P* Value**	**β**
*Frailty at Age 70 Years (Wave 1)* ^c^								
Neighborhood social deprivation	0.017	0.005, 0.029	0.007	0.223	0.010	−0.002, 0.022	0.109	0.144
Age, years	0.008	−0.002, 0.019	0.121	0.113	0.012	0.001, 0.022	0.027	0.174
Parental OSC III–V (referent: I and II)	−0.018	−0.044, 0.007	0.155	−0.246	−0.001	−0.024, 0.023	0.963	−0.008
IQ at age 11 years	−0.009	−0.021, 0.002	0.108	−0.126	−0.018	−0.029, 0.007	0.002	−0.263
Duration of education, years	−0.020	−0.034, −0.006	0.005	−0.269	0.001	−0.011, 0.013	0.907	0.010
Childhood smoking (referent: no)	0.011	−0.013, 0.035	0.363	0.146	0.002	−0.032, 0.037	0.893	0.035
Adult OSC III–V (referent: I and II)	0.007	−0.017, 0.030	0.583	0.088	−0.018	−0.040, 0.005	0.117	−0.265
Current smoking (referent: no)	−0.009	−0.052, 0.034	0.675	−0.122	0.004	−0.032, 0.040	0.818	0.062
*Frailty Progression Between Ages 70 and 82 Years (Waves 1–5)* ^d^								
Fixed effects								
Neighborhood social deprivation	0.017	0.005, 0.029	0.005	0.195	0.016	0.005, 0.027	0.004	0.196
Age, years	0.029	0.024, 0.034	0.000	0.333	0.027	0.023, 0.032	0.000	0.328
Parental OSC III–V (referent: I and II)	−0.014	−0.038, 0.010	0.260	−0.158	0.007	−0.014, 0.029	0.509	0.087
IQ at age 11 years	−0.009	−0.020, 0.001	0.092	−0.106	−0.022	−0.032, 0.012	0.000	−0.264
Duration of education, years	−0.018	−0.031, −0.005	0.008	−0.208	0.001	−0.010, 0.011	0.922	0.006
Childhood smoking (referent: no)	0.014	−0.008, 0.036	0.213	0.162	0.005	−0.025, 0.036	0.728	0.066
Adult OSC III–V (referent: I and II)	0.004	−0.019, 0.026	0.743	0.043	−0.023	−0.043, 0.003	0.023	−0.282
Current smoking (referent: no)	−0.003	−0.043, 0.038	0.899	−0.030	0.008	−0.025, 0.040	0.639	0.093
Neighborhood social deprivation × age	−0.001	−0.006, 0.004	0.645	−0.013	0.005	0.0004, 0.009	0.033	0.058
Random effects^e^								
Intercept	0.004 (0.062)				0.004 (0.059)			
Slope	0.001 (0.023)				0.000 (0.021)			
Residual	0.001 (0.037)				0.001 (0.038)			

Abbreviations: CI, confidence interval; IQ, intelligence quotient; OSC, occupational social class.

^a^ Regression coefficients (*b*) and 95% CIs are displayed; we also provide fully standardized coefficients (β) to aid interpretation. Continuous predictors are mean-centered and scaled.

^b^ The models adjusted for neighborhood social deprivation in the previous developmental period (i.e., young adulthood).

^c^ Models were based on linear regression.

^d^ Models were based on linear mixed-effects regression with random intercepts and slopes.

^e^ Values are expressed as variance (standard deviation).

Using linear mixed-effects regression, we investigated the effect of NSD × age interaction on frailty progression. We found that frailty trajectories were not associated with accumulated NSD in males (*b* = −0.001, 95% CI: −0.006, 0.004) (Table 2; Figure 3A). However, in females, a 1–standard-deviation higher score in mid- to late adulthood NSD was associated with a 0.005-point (95% CI: 0.0004, 0.009) change in Frailty Index score for each 1–standard-deviation increase in age (Table 2; Figure 3B), indicating widening NSD-based inequalities in frailty levels between ages 70 and 82 years (β = 0.058). Sex differences were confirmed in the total sample (*P* < 0.05). Finally, Johnson-Neyman intervals indicated that the association with NSD first materialized at age 70.5 years among females; but they also suggested that the association among males might diminish after the age of 81.2 years (Web Figure 3).

Sensitivity analyses extended and confirmed our findings. When we reran LARS model selection with regression residuals adjusted for all relevant DAG-based life-course–specific confounders, we found that the childhood (*P* = 0.006) and mid- to late adulthood (*P* = 0.022) sensitive periods were most appropriate models for males (*R*^2^ = 0.095). Among females, the mid- to late adulthood sensitive period remained as first selected (*R*^2^ = 0.003; *P* = 0.757). Stage 2 results were robust for frailty at baseline but became nonsignificant for frailty progression when the sample was restricted to LBC1936 participants who had lived in Edinburgh throughout their lives (Web Table 2) or when the temporal overlap between NSD and outcome assessment was eliminated (Web Table 3). The latter results stressed the importance of contemporaneous NSD exposure in frailty progression among females. When we replaced continuous exposures with tertiles, the findings confirmed the linear relationships (Web Table 4). Results on the dichotomized Frailty Index reinforced our findings: Higher accumulated NSD among males was associated with higher odds of being frail at age 70 years (odds ratio = 2.35, 95% CI: 1.40, 4.14). Among females, living in socially deprived neighborhoods during mid- to late adulthood was associated with higher odds of becoming frail during follow-up (odds ratio = 1.46, 95% CI: 1.03, 2.08) (Web Table 5). Finally, excluding cognitively compromised individuals at the time of address data collection did not change the findings (Web Table 6).

## DISCUSSION

Neighborhood social deprivation is an important predictor of frailty and frailty progression in old age, but the life-course relationship differs by sex. Using a structured modeling approach, we identified the relaxed accumulation hypothesis as best capturing the link between NSD and frailty among males, whereby the impact of living in socially deprived areas in childhood and mid- to late adulthood contributed to higher frailty in older age. Among females, higher NSD in mid- to late adulthood was associated with faster frailty progression: Divergent slopes first materialized around age 70.5 years.

Consistent with evidence relating to different health outcomes (22, 29, 32), neighborhood deprivation in mid- to late adulthood was associated with frailty and its progression, in addition to the impact of early exposures among males. Structural differences across neighborhoods, including social deprivation, can be linked to frailty via the stress pathway or through variations in providing collective resources and opportunities to residents to support their health and well-being (33–36). Living in socially deprived neighborhoods may affect health and frailty, via accumulation of stress over time. A recent investigation demonstrated that long-term exposure to deprived neighborhoods is associated with worse allostatic load—wear and tear on the body that is probably linked to chronic psychological stress exposure (36).

Advantaged neighborhoods may provide more opportunities to support health and well-being. Neighborhood-based social processes (e.g., social cohesion, social participation) protect against frailty by creating and maintaining social connections and support networks and by buffering stress (33, 34, 36, 37). Availability of recreational and cultural facilities has been associated with slower age-related decline (38). While perceiving residential areas as unsafe (34) or deteriorating (33) is associated with frailty, probably via higher stress levels, avoidance behavior, and maladaptive coping mechanisms (33, 39), greater access to green space could stimulate engagement in physical and social activities, improving frailty status (40). However, understanding why neighborhood-based inequalities in frailty persisted among males during almost the entire follow-up period of the study (ages 70–82 years) but only first materialized at age 70.5 years for females requires further exploration; it might be linked to the role of neighborhood resources and stressors across the life course.

In addition to mid- to late adulthood exposure, deprivation in childhood was linked to frailty among males. Childhood is a formative developmental period; living in deprived neighborhoods at a young age can adversely affect health in childhood and early adulthood (41), potentially through disruption of stress regulation (42) or through alterations to the epigenome (43). Moreover, early-life exposure can predict subsequent adverse (neighborhood) exposures, as described in the chain-of-risk hypothesis (22). Sex differences in the early-life context may be linked to higher susceptibility and exposure to environmental influences among boys, partly related to decreased parental supervision and stronger neighborhood influences on future employment aspirations (44). Gendered early-life neighborhood experiences were probably even more distinct in the first half of the 20th century, with greater expectation of girls’ undertaking household domestic work while boys were more engaged in activities in their wider neighborhood (45).

To our knowledge, this is the first study to have explored the impact of neighborhood context across most of the life course on frailty and frailty progression. We utilized information on NSD covering the period from birth to late adulthood, repeated measures of frailty based on 30 health deficits, and key life-course confounders (e.g., childhood intelligence). Applying the novel structured life-course modeling approach reduced the risk of bias arising from simultaneously testing competing theoretical models and enabled us to choose parsimonious life-course models, without overinflating effect-size estimates during variable selection (26) or biasing hypothesis testing (28).

Still, our study had several limitations. First, the historical measure of NSD was only available for the City of Edinburgh, which led to a modest sample size due to missing exposure data for participants residing elsewhere. Second, despite residential addresses’ being recalled with adequate accuracy in life grid questionnaires (16), retrospective data collection is prone to recall bias (12). Third, we were constrained by the availability of historical area-level information and the spatial scale reported in official records. Information on NSD was aggregated at the ward level and was limited to a small number of indicators that were not consistently available throughout the study. Consequently, NSD was measured with 2 strongly correlated but distinct constructs (12). Moreover, neighborhood deprivation at the ward level is unlikely to precisely overlap with participants’ self-defined neighborhoods, and utilizing larger geographic units, such as wards, probably underestimates area effects (46). Ward boundaries in 1961 differed from contemporary wards due to substantial change in spatial delineation; still, it was the only common spatial resolution available to us and was needed to handle missing area-level data (12). Fourth, selection and survival bias probably affected our findings. Population-level data derived from the Scottish Mental Survey 1947 suggest that LBC1936 participants had higher childhood intelligence than the population average (47), and thus they were more likely to live longer (48). Our analytical sample was similar to the full LBC1936 cohort in terms of socioeconomic indicators and childhood IQ, but it included healthier and younger participants. Furthermore, residential histories were collected after wave 3, when participants were in their late 70s, limiting the generalizability of findings and introducing survival bias. Last, because structural life-course modeling is not available for outcomes with repeated measurements, we conducted LARS variable selection only for frailty at age 70 years. Future methodological developments would usefully take into account changes in outcome levels.

In conclusion, our findings showed, in an Edinburgh-based sample, that NSD across the life course matters for frailty in older adulthood. While the impact of deprivation likely accumulates among males, with childhood and mid- to late adulthood being pertinent, among females, living in deprived areas during the second part of life might be more relevant. Given the above limitations, future research could usefully replicate our findings in large-scale longitudinal studies with more diverse populations and could explore specific neighborhood mechanisms (e.g., social, environmental, geographic, institutional) (49) that link structural area differences to age-related decline. Understanding causal routes by which individuals growing up, living, and aging in different contexts across the life course become frail and identifying vulnerable groups may have policy implications. Having access to good-quality neighborhoods from childhood onwards and placing multimodal frailty interventions (38) in deprived areas may support healthy aging by preventing and slowing age-related decline. Integration of the life-course framework into community-based policies presents an opportunity to maintain health and well-being in the context of global population aging.

## Supplementary Material

Web_Material_kwac134
