# Supplementary material for: Association of Life-Course Neighborhood Deprivation With Frailty and Frailty Progression From Ages 70 to 82 Years in the Lothian Birth Cohort 1936
Source: Am J Epidemiol. 2022 Jul 27;191(11):1856–66. doi: 10.1093/aje/kwac134 (PMC9626928; doi:10.1093/aje/kwac134)
Supplement: Web_Material_kwac134 [file web_material_kwac134.pdf]

Web Material

Association of Life-Course Neighborhood Deprivation With Frailty and Frailty Progression From Ages 70 to 82 Years in the Lothian Birth Cohort 1936

Gergő Baranyi, Miles Welstead, Janie Corley, Ian J. Deary, Graciela Muniz-Terrera, Paul Redmond, Niamh Shortt, Adele M. Taylor, Catharine Ward Thompson, Simon R. Cox, and Jamie Pearce

<u>Contents</u>

Web Figure 1. Heatmap depicting Pearson's correlation coefficients between neighborhood social deprivation scores across participants' life course

Web Table 1. Health deficits included in the Frailty Index

Web Figure 2. Directed acyclic graph presenting associations between neighborhood social deprivation, frailty and life course covariates, including selective migration processes linked to earlier life health and socioeconomic conditions.

Web Figure 3. Johnson-Neyman plots of neighborhood social deprivation slopes and their confidence bands depicting regions of significance conditional on age

Web Table 2. Main models among participants remaining in Edinburgh throughout their lives

Web Table 3. Main models based on non-overlapping exposure and outcome measurements

Web Table 4. Main models with tertiles of neighborhood social deprivation

Web Table 5. Main models with dichotomous frailty measurement

Web Table 6. Main models after excluding participants with cognitive impairment

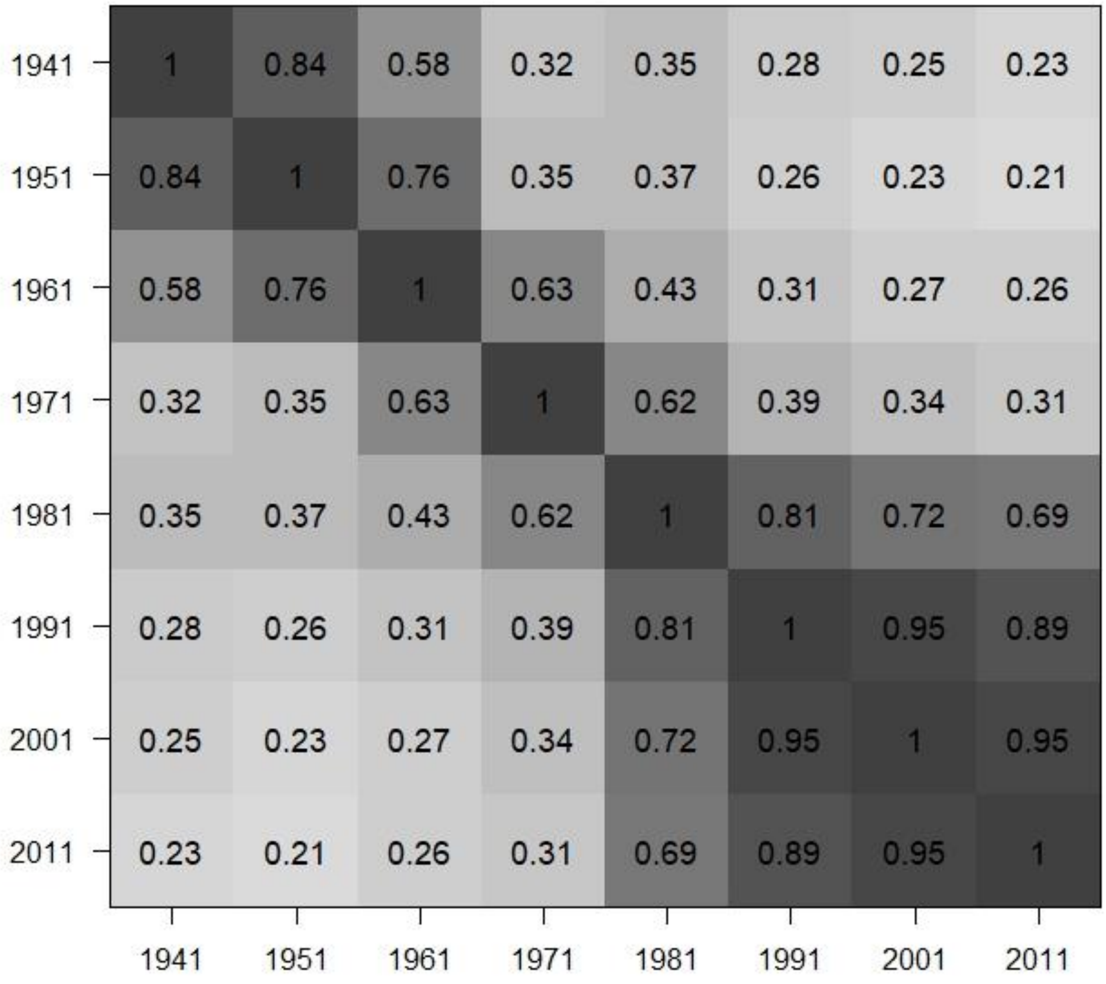

Web Figure 1. Heatmap depicting Pearson's correlation coefficients between neighborhood social deprivation scores across participants' life course. Correlation coefficients was calculated only for participants providing Edinburgh-based addresses at least once for each decades of their lives (*n* = 247).

Web Table 1. Health deficits included in the Frailty Index.[a]

Item	Coding	Notes
Systolic blood pressure	<5th percentile (1), 5th-20th percentile (0.5), >20th percentile (0)	Recommended technique[1]
Diabetes (self-reported)	Yes (1) or No (0)	Already binary variable
High Cholesterol (self-reported)	Yes (1) or No (0)	Already binary variable
Heart problems (self-reported)	Yes (1) or No (0)	Already binary variable
Stroke or mini stroke (self-reported)	Yes (1) or No (0)	Already binary variable
Crampy pains in calves (self-reported)	Yes (1) or No (0)	Already binary variable
Blood circulation issues (self-reported)	Yes (1) or No (0)	Already binary variable
Thyroid Disorder (self-reported)	Yes (1) or No (0)	Already binary variable
Cancer (self-reported)	Yes (1) or No (0)	Already binary variable
Parkinson's disease (self-reported)	Yes (1) or No (0)	Already binary variable
Dementia (self-reported)	Yes (1) or No (0)	Already binary variable
Arthritis (self-reported)	Yes (1) or No (0)	Already binary variable
Any other chronic disease (self-reported)	Yes (1) or No (0)	Already binary variable
Polypharmacy (self-reported)	>4 medications (1), ≤4 medications (0)	Recommended technique[2]
Body Mass Index	18.5 to <25 (0), 25 to <30 (0.5), <18.5 or >equal to 30 (1)	Recommended technique[3]
6 m walk time (gait speed)	>10 seconds or physically unable (1), <10 seconds (0)	Recommended technique[4]
Able to stand up from a chair	Yes (1) or No (0)	Already binary variable
Grip strength (strongest hand and stratified by sex and BMI)	<5th percentile (1), 5th-20th percentile (0.5), >20th percentile (0)	Recommended technique[1]
Townsend Disability Scale	<5th percentile (1), 5th-20th percentile (0.5), >20th percentile (0)	Recommended technique[1]
Peak Expiratory Flow rate (stratified by sex)	<5th percentile (1), 5th-20th percentile (0.5), >20th percentile (0)	Recommended technique[1]
Forced expiratory volume (stratified by sex)	<5th percentile (1), 5th-20th percentile (0.5), >20th percentile (0)	Recommended technique[1]
Depression (measured in the HADS)	11 -21 (1), 8 – 10 (0.5), 0 – 7 (0)	Recommended technique[5]
Anxiety (measured in the HADS)	11 -21 (1), 8 – 10 (0.5), 0 – 7 (0)	Recommended technique[5]
Mini-Mental State Examination	<10 (1), 11-17 (0.75), 18 – 20 (0.5), 20 – 24 (0.25), >24 (0)	Recommended technique[6]
Digit Symbol (measured in WAIS-III)	<5th percentile (1), 5th-20th percentile (0.5), >20th percentile (0)	Recommended technique[1]
Block Design (measured in WAIS-III)	<5th percentile (1), 5th-20th percentile (0.5), >20th percentile (0)	Recommended technique[1]
Verbal Fluency	<5th percentile (1), 5th-20th percentile (0.5), >20th percentile (0)	Recommended technique[1]
Matrix Reasoning (measured in WAIS-III)	<5th percentile (1), 5th-20th percentile (0.5), >20th percentile (0)	Recommended technique[1]
Reaction time test	<5th percentile (1), 5th-20th percentile (0.5), >20th percentile (0)	Recommended technique[1]
Delayed recall	<5th percentile (1), 5th-20th percentile (0.5), >20th percentile (0)	Recommended technique[1]

Abbreviations: BMI, body mass index; HADS, Hospital Anxiety and Depression scale; WAIS, Wechsler Adult Intelligence Scale.

[a]Table is based on Table A1 in Welstead et al. 2020.[7]

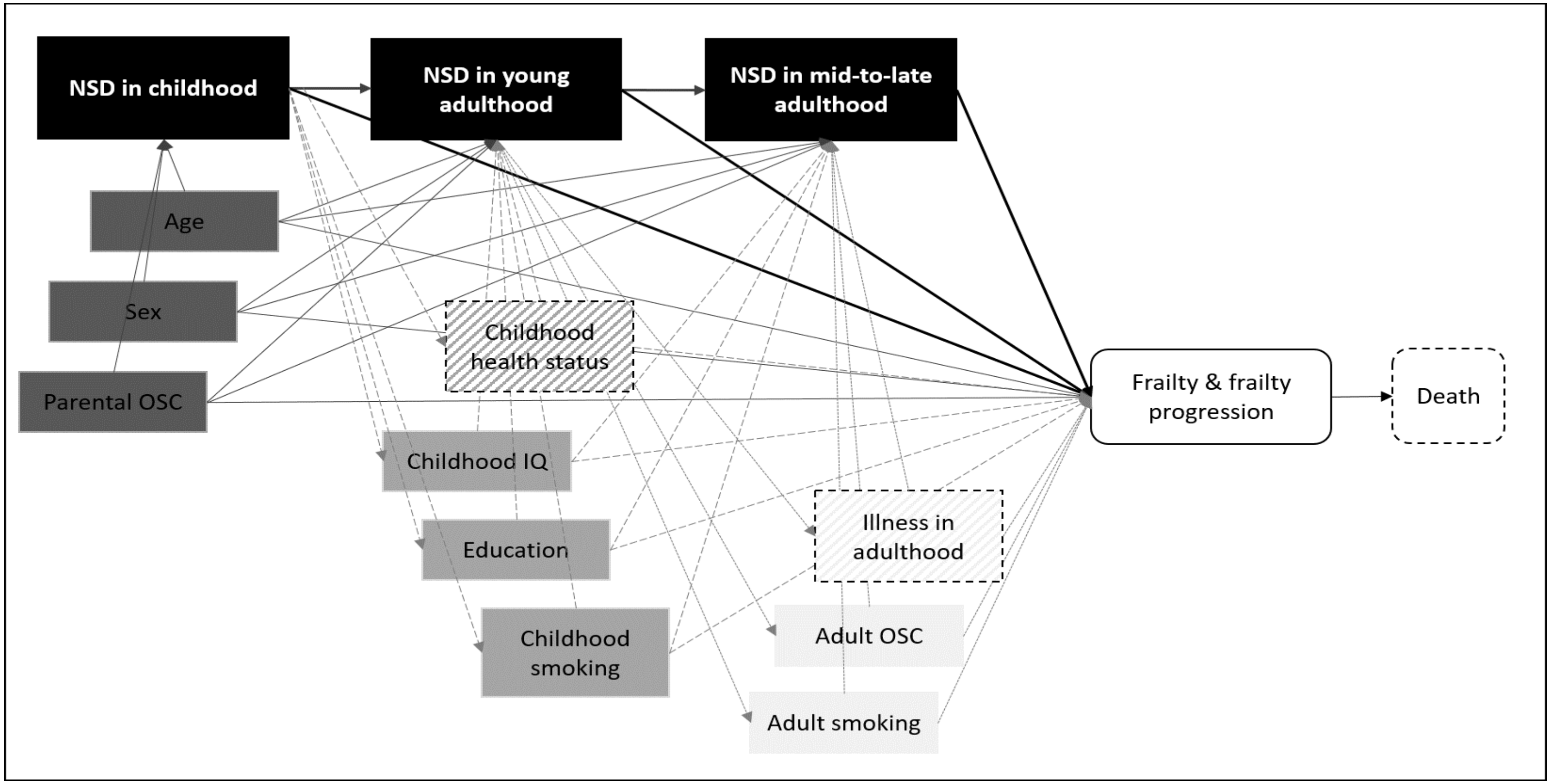

Web Figure 2. Directed acyclic graph presenting associations between neighborhood social deprivation, frailty and life course covariates, including selective migration processes linked to earlier life health and socioeconomic conditions. Exposure to NSD in childhood is associated with age, sex and parental OSC: key confounders for childhood NSD and frailty. However, they are also associated with subsequent neighborhood exposures, indicating that these variables have to be carried over as confounders in later life models. Similarly, childhood variables (e.g. childhood health, education), influenced by early life neighborhood exposures, are confounders for NSD in adulthood and frailty, as they may relate to neighborhood selection from young adulthood onwards. Finally, covariates during adulthood (e.g. adult OSC) may confound the NSD in mid-to-late adulthood and frailty relationship. *Note*: Links between covariates are not shown for simplicity. The graph presents measured (solid line) and unmeasured (dashed line) covariates, and an unmeasured competing event (death). Differently coloured covariates and arrows indicate time-specific confounding (i.e. dark grey colour/solid line – childhood onwards; medium grey colour/dashed line – young adulthood onwards; light grey colour/dotted line – mid-to-late adulthood). NSD, neighborhood social deprivation; OSC, occupational social class.

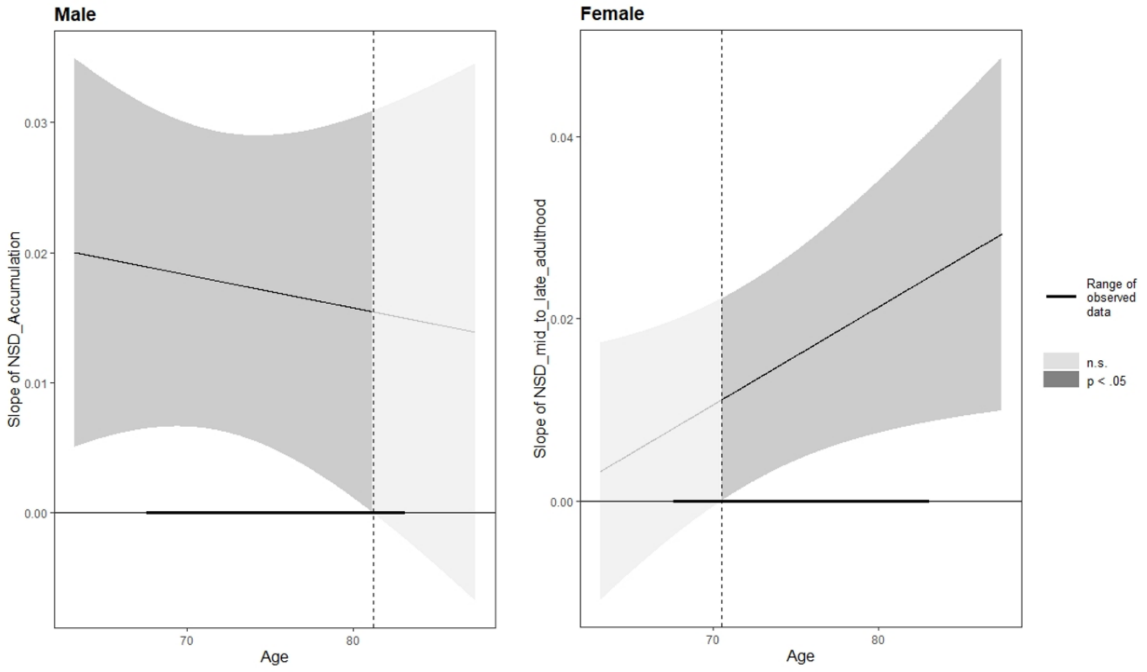

Web Figure 3. Johnson-Neyman plots of neighborhood social deprivation slopes and their confidence bands depicting regions of significance conditional on age. Darker grey areas indicate age intervals with significant, lighter grey areas with non-significant neighborhood social deprivation and fraily associations; dark horizontal lines represent the range of observed data. Dotted verical lines depict the thresholds where significance changes in the male (81.23 years old) and female (70.48 years old) subsamples. Calculations are based on most appropriate life-course models for male (i.e. accumulation) and female (i.e. mid-to-late adulthood sensitive period) participants. Intervals were adjusted for false discovery rate. Abbreviation: n.s., not significant; NSD, neighborhood social deprivation.

Web Table 2. Main models among participants remaining in Edinburgh throughout their lives, Lothian Birth Cohort 1936, 1936–2019[a]

	Male (*n*=127) Accumulation				Female (*n*=120) Mid-to-late adulthood sensitive period[b]			
	b	95% CI	*P*-Value	*β*	*b*	95% CI	*P*-Value	*β*
**Frailty at age 70 (wave 1)[c]**								
Neighborhood social deprivation	0.015	0.001, 0.030	0.034	0.206	0.013	-0.002, 0.027	0.081	0.189
Age	0.005	-0.007, 0.018	0.406	0.072	0.015	0.003, 0.027	0.015	0.221
Parental occupational social class (ref: I & II)	-0.012	-0.044, 0.019	0.437	-0.165	-0.008	-0.036, 0.021	0.601	-0.114
IQ at age 11	-0.009	-0.022, 0.004	0.182	-0.116	-0.020	-0.032, -0.007	0.002	-0.298
Years spent in education	-0.022	-0.039, -0.006	0.009	-0.295	0.004	-0.009, 0.017	0.542	0.059
Childhood smoking (ref: no)	0.004	-0.023, 0.031	0.754	0.057	0.005	-0.030, 0.040	0.772	0.077
Adult occupational social class (ref: I & II)	0.004	-0.023, 0.032	0.761	0.057	-0.015	-0.039, 0.009	0.215	-0.228
Current smoking (ref: no)	0.005	-0.045, 0.055	0.841	0.068	0.006	-0.036, 0.048	0.771	0.092
**Frailty progression between age 70 and 82 (wave 1-5)[d]**								
Fixed effects								
Neighborhood social deprivation	0.017	0.004, 0.030	0.012	0.193	0.018	0.005, 0.030	0.006	0.215
Age	0.029	0.024, 0.035	0.000	0.333	0.029	0.024, 0.034	0.000	0.353
Parental occupational social class (ref: I & II)	-0.005	-0.033, 0.023	0.739	-0.055	-0.004	-0.029, 0.020	0.731	-0.052
IQ at age 11	-0.008	-0.019, 0.003	0.173	-0.091	-0.026	-0.037, -0.015	0.000	-0.316
Years spent in education	-0.020	-0.035, -0.005	0.009	-0.232	0.003	-0.007, 0.014	0.571	0.037
Childhood smoking (ref: no)	0.007	-0.017, 0.031	0.576	0.079	0.010	-0.019, 0.040	0.491	0.126
Adult occupational social class (ref: I & II)	0.001	-0.024, 0.026	0.955	0.008	-0.025	-0.045, -0.004	0.019	-0.302
Current smoking (ref: no)	0.013	-0.032, 0.057	0.585	0.143	0.005	-0.030, 0.041	0.772	0.064
Neighborhood social deprivation*Age	0.001	-0.004, 0.007	0.638	0.015	0.004	-0.001, 0.009	0.129	0.050
Random effects								
Intercept	0.004 (0.061)[e]				0.003 (0.055)[e]			
Slope	0.001 (0.024)[e]				0.000 (0.022)[e]			
Residuals	0.001 (0.038)[e]				0.001 (0.038)[e]			

Abbreviations: CI, confidence interval.

[a] Regression coefficients (*b*) and 95% CI are displayed; we also provide fully standardized coefficients (*β*) to aid interpretation. Continuous predictors are mean-centered and scaled.

[b] Models are adjusted for neighborhood social deprivation in the previous developmental period (i.e. young adulthood).

[c] Models are based on linear regression.

[d] Models are based on linear mixed-effects regression with random intercepts and slopes.

[e] Values are expressed as variance (standard deviation).

Web Table 3. Main models based on non-overlapping exposure and outcome measurements, Lothian Birth Cohort 1936, 1936–2019[a]

	**Male (*n*=161)<br>Accumulation**				**Female (*n*=161)<br>Mid-to-late adulthood sensitive period[b]**			
	b	95% CI	*P*-Value	*β*	*b*	95% CI	*P*-Value	*β*
**Frailty at age 70 (wave 1)[c]**								
Neighborhood social deprivation	0.017	0.004, 0.029	0.007	0.220	0.010	-0.002, 0.022	0.100	0.150
Age	0.009	-0.002, 0.019	0.113	0.115	0.012	0.002, 0.022	0.025	0.177
Parental occupational social class (ref: I & II)	-0.019	-0.044, 0.007	0.153	-0.247	-0.001	-0.025, 0.023	0.933	-0.015
IQ at age 11	-0.009	-0.021, 0.002	0.108	-0.126	-0.018	-0.029, -0.007	0.002	-0.263
Years spent in education	-0.020	-0.034, -0.006	0.005	-0.270	0.001	-0.011, 0.013	0.816	0.021
Childhood smoking (ref: no)	0.011	-0.013, 0.035	0.364	0.146	0.002	-0.032, 0.036	0.900	0.032
Adult occupational social class (ref: I & II)	0.007	-0.017, 0.031	0.580	0.089	-0.018	-0.041, 0.004	0.113	-0.268
Current smoking (ref: no)	-0.009	-0.052, 0.034	0.688	-0.117	0.004	-0.032, 0.040	0.835	0.056
**Frailty progression between age 70 and 82 (wave 1-5)[d]**								
Fixed effects								
Neighborhood social deprivation	0.017	0.006, 0.029	0.004	0.197	0.016	0.005, 0.027	0.006	0.193
Age	0.029	0.024, 0.034	0.000	0.333	0.027	0.023, 0.032	0.000	0.328
Parental occupational social class (ref: I & II)	-0.014	-0.038, 0.010	0.257	-0.159	0.007	-0.015, 0.028	0.535	0.082
IQ at age 11	-0.009	-0.020, 0.001	0.091	-0.107	-0.022	-0.032, -0.012	0.000	-0.265
Years spent in education	-0.018	-0.032, -0.005	0.008	-0.209	0.001	-0.009, 0.012	0.822	0.015
Childhood smoking (ref: no)	0.014	-0.008, 0.036	0.214	0.162	0.005	-0.025, 0.036	0.731	0.065
Adult occupational social class (ref: I & II)	0.004	-0.019, 0.026	0.743	0.043	-0.024	-0.044, -0.004	0.022	-0.285
Current smoking (ref: no)	-0.002	-0.043, 0.038	0.918	-0.024	0.007	-0.025, 0.040	0.659	0.088
Neighborhood social deprivation*Age	-0.001	-0.006, 0.004	0.737	-0.009	0.004	-0.00002, 0.009	0.053	0.053
Random effects								
Intercept		0.004 (0.062)[e]				0.004 (0.059)[e]		
Slope		0.001 (0.023)[e]				0.000 (0.021)[e]		
Residuals		0.001 (0.037)[e]				0.001 (0.039)[e]		

Abbreviations: CI, confidence interval.

[a] Regression coefficients (*b*) and 95% CI are displayed; we also provide fully standardized coefficients (*β*) to aid interpretation. Continuous predictors are mean-centered and scaled.

[b] Models are adjusted for neighborhood social deprivation in the previous developmental period (i.e. young adulthood).

[c] Models are based on linear regression.

[d] Models are based on linear mixed-effects regression with random intercepts and slopes.

[e] Values are expressed as variance (standard deviation).

Web Table 4. Main models with tertiles of neighborhood social deprivation, Lothian Birth Cohort 1936, 1936–2019[a]

	**Male (*n*=161) Accumulation**				**Female (*n*=162) Mid-to-late adulthood sensitive period[b]**			
	b	95% CI	*P*-Value	*β*	*b*	95% CI	*P*-Value	*β*
**Frailty at age 70 (wave 1)[c]**								
Neighborhood social deprivation (ref: Low)								
Medium	0.014	-0.014, 0.042	0.318	0.186	0.018	-0.008, 0.044	0.172	0.268
High	0.040	0.011, 0.069	0.007	0.532	0.007	-0.021, 0.036	0.607	0.110
Age	0.009	-0.002, 0.020	0.108	0.117	0.012	0.002, 0.023	0.025	0.178
Parental occupational social class (ref: I & II)	-0.019	-0.044, 0.007	0.156	-0.248	0.002	-0.022, 0.026	0.870	0.029
IQ at age 11	-0.009	-0.021, 0.003	0.132	-0.119	-0.019	-0.030, -0.008	0.001	-0.276
Years spent in education	-0.022	-0.036, -0.008	0.002	-0.291	-0.000	-0.012, 0.011	0.941	-0.006
Childhood smoking (ref: no)	0.009	-0.015, 0.033	0.448	0.122	0.005	-0.029, 0.040	0.758	0.080
Adult occupational social class (ref: I & II)	0.005	-0.020, 0.029	0.709	0.061	-0.016	-0.039, 0.007	0.162	-0.237
Current smoking (ref: no)	-0.009	-0.052, 0.034	0.677	-0.122	0.001	-0.035, 0.038	0.953	0.016
**Frailty progression between age 70 and 82 (wave 1-5)[d]**								
Fixed effects								
Neighborhood social deprivation (ref: Low)								
Medium	0.021	-0.006, 0.048	0.124	0.245	0.031	0.006, 0.055	0.016	0.369
High	0.035	0.007, 0.062	0.017	0.395	0.032	0.005, 0.058	0.019	0.384
Age	0.029	0.021, 0.037	0.000	0.332	0.019	0.011, 0.027	0.000	0.232
Parental occupational social class (ref: I & II)	-0.014	-0.039, 0.010	0.251	-0.163	0.010	-0.012, 0.031	0.376	0.117
IQ at age 11	-0.008	-0.019, 0.002	0.130	-0.097	-0.023	-0.033, -0.013	0.000	-0.275
Years spent in education	-0.021	-0.034, -0.008	0.002	-0.237	-0.001	-0.011, 0.010	0.918	-0.007
Childhood smoking (ref: no)	0.013	-0.010, 0.035	0.260	0.149	0.008	-0.023, 0.039	0.609	0.097
Adult occupational social class (ref: I & II)	0.003	-0.020, 0.026	0.798	0.034	-0.022	-0.042, -0.002	0.032	-0.266
Current smoking (ref: no)	-0.001	-0.042, 0.039	0.946	-0.016	0.004	-0.029, 0.037	0.804	0.050
Neighborhood social deprivation *Age (ref: Low*Age)								
Medium*Age	0.005	-0.007, 0.017	0.400	0.057	0.007	-0.004, 0.017	0.233	0.080
High*Age	-0.004	-0.015, 0.007	0.483	-0.046	0.016	0.005, 0.027	0.005	0.190
Random effects								
Intercept		0.004 (0.063)[e]				0.004 (0.059)[e]		
Slope		0.001 (0.023)[e]				0.000 (0.020)[e]		
Residuals		0.001 (0.037)[e]				0.001 (0.039)[e]		

Abbreviations: CI, confidence interval.

[a] Regression coefficients (*b*) and 95% CI are displayed; we also provide fully standardized coefficients (*β*) to aid interpretation. Continuous predictors are mean-centered and scaled.

[b] Models are adjusted for neighborhood social deprivation in the previous developmental period (i.e., young adulthood).
[c] Models are based on linear regression.
[d] Models are based on linear mixed-effects regression with random intercepts and slopes.
[e] Values are expressed as variance (standard deviation).

Web Table 5. Main models with dichotomous frailty measurement, Lothian Birth Cohort 1936, 1936–2019[a]

	**Male (*n*=161) Accumulation**			**Female (*n*=162) Mid-to-late adulthood sensitive period[b]**		
	OR	95% CI	*P*-Value	OR	95% CI	*P*-Value
**Frailty at age 70 (wave 1)[c]**						
Neighborhood social deprivation	2.35	1.40-4.14	0.002	1.21	0.76-1.92	0.417
Age	1.41	0.89-2.29	0.152	1.24	0.83-1.85	0.296
Parental occupational social class (ref: I & II)	0.82	0.25-2.72	0.741	0.95	0.40-2.36	0.915
IQ at age 11	0.49	0.29-0.78	0.004	0.64	0.42-0.95	0.030
Years spent in education	0.49	0.20-1.06	0.091	1.03	0.66-1.60	0.883
Childhood smoking (ref: no)	0.82	0.32-2.02	0.664	0.66	0.14-2.32	0.546
Adult occupational social class (ref: I & II)	0.66	0.25-1.72	0.396	0.52	0.22-1.20	0.133
Current smoking (ref: no)	0.40	0.05-2.36	0.345	1.97	0.56-6.38	0.267
**Frailty progression between age 70 and 82 (wave 1-5)[d]**						
Fixed effects						
Neighborhood social deprivation	3.03	1.43-6.43	0.004	2.14	1.13-4.07	0.02
Age	3.63	2.26-5.82	<0.001	3.42	2.28-5.11	<0.001
Parental occupational social class (ref: I & II)	0.31	0.08-1.22	0.095	1.36	0.39-4.81	0.632
IQ at age 11	0.41	0.22-0.74	0.003	0.40	0.21-0.73	0.003
Years spent in education	0.38	0.16-0.87	0.022	1.02	0.55-1.90	0.943
Childhood smoking (ref: no)	1.06	0.31-3.66	0.888	2.27	0.40-12.85	0.352
Adult occupational social class (ref: I & II)	0.70	0.21-2.29	0.552	0.18	0.05-0.64	0.008
Current smoking (ref: no)	0.35	0.03-3.70	0.384	2.28	0.36-14.48	0.381
Neighborhood social deprivation *Age	0.85	0.54-1.33	0.469	1.46	1.03-2.08	0.034
Random effects						
Intercept, variance (SD)	9.94 (3.15)[e]			8.10 (2.85)[e]		
Slope, variance (SD)	2.10 (1.45)[e]			0.96 (0.98)[e]		

Abbreviations: CI, confidence interval; OR, Odds Ratio.

[a] ORs and 95% CI are displayed; continuous predictors are mean-centered and scaled.

[b] Models are adjusted for neighborhood social deprivation in the previous developmental period (i.e. young adulthood).

[c] Models are based on logistic regression.

[d] Models are based on mixed-effects logistic regression with random intercepts and slopes.

[e] Values are expressed as variance (standard deviation).

Web Table 6. Main models after excluding participants with cognitive impairment, Lothian Birth Cohort 1936, 1936–2019[a]

	Male (*n*=153) Accumulation				Female (*n*=160) Mid-to-late adulthood sensitive period[b]			
	b	95% CI	*P*-Value	*β*	*b*	95% CI	*P*-Value	*β*
**Frailty at age 70 (wave 1)[c]**								
Neighborhood social deprivation	0.019	0.006, 0.031	0.003	0.249	0.010	-0.002, 0.022	0.107	0.146
Age	0.008	-0.004, 0.019	0.182	0.101	0.011	0.001, 0.022	0.036	0.167
Parental occupational social class (ref: I & II)	-0.015	-0.041, 0.011	0.253	-0.201	0.000	-0.024, 0.024	0.998	0.000
IQ at age 11	-0.007	-0.019, 0.005	0.256	-0.091	-0.018	-0.029, -0.007	0.002	-0.262
Years spent in education	-0.021	-0.035, -0.007	0.005	-0.279	0.000	-0.012, 0.012	0.961	0.004
Childhood smoking (ref: no)	0.010	-0.014, 0.035	0.408	0.138	0.002	-0.032, 0.037	0.900	0.032
Adult occupational social class (ref: I & II)	0.005	-0.019, 0.029	0.676	0.069	-0.018	-0.040, 0.005	0.123	-0.261
Current smoking (ref: no)	-0.009	-0.052, 0.034	0.683	-0.119	0.004	-0.032, 0.041	0.812	0.065
**Frailty progression between age 70 and 82 (wave 1-5)[d]**								
Fixed effects								
Neighborhood social deprivation	0.018	0.006, 0.029	0.004	0.203	0.017	0.006, 0.028	0.004	0.200
Age	0.028	0.023, 0.032	0.000	0.319	0.027	0.023, 0.031	0.000	0.326
Parental occupational social class (ref: I & II)	-0.010	-0.035, 0.014	0.405	-0.120	0.008	-0.013, 0.030	0.449	0.100
IQ at age 11	-0.006	-0.017, 0.005	0.252	-0.075	-0.022	-0.032, -0.012	0.000	-0.267
Years spent in education	-0.019	-0.032, -0.005	0.008	-0.215	-0.000	-0.011, 0.010	0.983	-0.001
Childhood smoking (ref: no)	0.015	-0.008, 0.038	0.207	0.173	0.005	-0.026, 0.036	0.742	0.062
Adult occupational social class (ref: I & II)	0.002	-0.021, 0.025	0.846	0.026	-0.023	-0.043, -0.003	0.025	-0.281
Current smoking (ref: no)	-0.003	-0.043, 0.037	0.877	-0.037	0.008	-0.024, 0.041	0.622	0.099
Neighborhood social deprivation *Age	-0.002	-0.007, 0.003	0.366	-0.025	0.005	0.001, 0.010	0.020	0.063
Random effects								
Intercept,		0.004 (0.062)[e]				0.003 (0.059)[e]		
Slope		0.000 (0.021)[e]				0.000 (0.020)[e]		
Residuals		0.001 (0.038)[e]				0.001 (0.039)[e]		

Abbreviations: CI, confidence interval.

[a] Cognitive impairment was defined as either having a diagnosis of dementia or scoring <24 points in the Mini Mental State Examination. Regression coefficients (*b*) and 95% CI are displayed; we also provide fully standardized coefficients (*β*) to aid interpretation. Continuous predictors are mean-centered and scaled.

[b] Models are adjusted for neighborhood social deprivation in the previous developmental period (i.e., young adulthood).

[c] Models are based on linear regression.

[d] Models are based on linear mixed-effects regression with random intercepts and slopes.

[e] Values are expressed as variance (standard deviation).

REFERENCES

1. Theou O, O'Connell MDL, King-Kallimanis BL, O'Halloran AM, Rockwood K, Kenny RA. Measuring frailty using self-report and test-based health measures. *Age Ageing* 2015;44:471–477.
2. Theou O, Brothers TD, Mitnitski A, Rockwood K. Operationalization of frailty using eight commonly used scales and comparison of their ability to predict all-cause mortality. *J Am Geriatr Soc* 2013;61:1537–1551.
3. Chamberlain AM, St. Sauver JL, Jacobson DJ, et al. Social and behavioural factors associated with frailty trajectories in a population-based cohort of older adults. *BMJ Open* 2016;6:e011410.
4. Hoogendijk EO, Theou O, Rockwood K, Onwuteaka-Philipsen BD, Deeg DJH, Huisman M. Development and validation of a frailty index in the Longitudinal Aging Study Amsterdam. *Aging Clin Exp Res* 2017;29:927–933.
5. Zigmond AS, Snaith RP. The Hospital Anxiety and Depression Scale. *Acta Psychiatr Scand* 1983;67:361–70.
6. Searle SD, Mitnitski A, Gahbauer EA, Gill TM, Rockwood K. A standard procedure for creating a frailty index. *BMC Geriatr* 2008;8:24.
7. Welstead M, Muniz-Terrera G, Russ TC, et al. Inflammation as a risk factor for the development of frailty in the Lothian Birth Cohort 1936. *Exp Gerontol* 2020;139:111055.
